# *N*-(3,5-Dimethyl­phen­yl)succinamic acid

**DOI:** 10.1107/S1600536810053055

**Published:** 2010-12-24

**Authors:** B. S. Saraswathi, Sabine Foro, B. Thimme Gowda

**Affiliations:** a Department of Chemistry, Mangalore University, Mangalagangotri 574 199, Mangalore, India; b Institute of Materials Science, Darmstadt University of Technology, Petersenstrasse 23, D-64287 Darmstadt, Germany

## Abstract

In the title compound, C_12_H_15_NO_3_, the N—H and C=O bonds are *anti* to each other. The C=O and O—H bonds of the acid group display an anti­periplanar orientation relative to each other. The crystal packing features a three-dimensional network of molecules held together by O—H⋯O and N—H⋯O hydrogen bonds.

## Related literature

For our study of the effect of ring and side-chain substitutions on the crystal structures of anilides, see: Gowda *et al.* (2009 ▶ 2010*a* ▶,*b* ▶). For modes of inter­linking carb­oxy­lic acids by hydrogen bonds, see: Leiserowitz (1976 ▶). The packing of mol­ecules involving dimeric hydrogen-bonded association of each carboxyl group with a centrosymmetrically related neighbor has also been observed, see: Jagannathan *et al.* (1994 ▶).
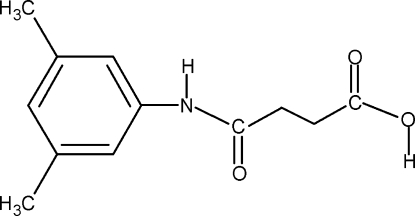


## Experimental

### 

#### Crystal data

C_12_H_15_NO_3_
*M* *_r_* = 221.25
Monoclinic, 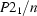

*a* = 14.346 (2) Å
*b* = 5.0225 (9) Å
*c* = 17.860 (3) Å
β = 112.00 (2)°
*V* = 1193.2 (3) Å^3^
*Z* = 4
Mo *K*α radiation
μ = 0.09 mm^−1^
*T* = 293 K
0.45 × 0.08 × 0.05 mm

#### Data collection

Oxford Diffraction Xcalibur diffractometer with a Sapphire CCD detector
Absorption correction: multi-scan (*CrysAlis RED*; Oxford Diffraction, 2009 ▶) *T*_min_ = 0.961, *T*_max_ = 0.996
4452 measured reflections
2419 independent reflections
1593 reflections with *I* > 2σ(*I*)
*R*_int_ = 0.019

#### Refinement

*R*[*F* ^2^ > 2σ(*F* ^2^)] = 0.054
*wR*(*F* ^2^) = 0.147
*S* = 1.03
2419 reflections
153 parameters
H atoms treated by a mixture of independent and constrained refinement
Δρ_max_ = 0.20 e Å^−3^
Δρ_min_ = −0.18 e Å^−3^

### 

Data collection: *CrysAlis CCD* (Oxford Diffraction, 2009 ▶); cell refinement: *CrysAlis RED* (Oxford Diffraction, 2009 ▶); data reduction: *CrysAlis RED*; program(s) used to solve structure: *SHELXS97* (Sheldrick, 2008 ▶); program(s) used to refine structure: *SHELXL97* (Sheldrick, 2008 ▶); molecular graphics: *PLATON* (Spek, 2009 ▶); software used to prepare material for publication: *SHELXL97*.

## Supplementary Material

Crystal structure: contains datablocks I, global. DOI: 10.1107/S1600536810053055/bt5440sup1.cif

Structure factors: contains datablocks I. DOI: 10.1107/S1600536810053055/bt5440Isup2.hkl

Additional supplementary materials:  crystallographic information; 3D view; checkCIF report

## Figures and Tables

**Table 1:** Hydrogen-bond geometry (Å, °)

*D*—H⋯*A*	*D*—H	H⋯*A*	*D*⋯*A*	*D*—H⋯*A*
N1—H1*N*⋯O2^i^	0.88 (2)	2.01 (2)	2.881 (2)	171 (2)
O3—H3*O*⋯O1^ii^	0.86 (3)	1.77 (3)	2.630 (2)	172 (3)

Symmetry codes: (i) 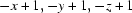; (ii) 
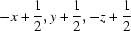
.

**Fig. 1.**
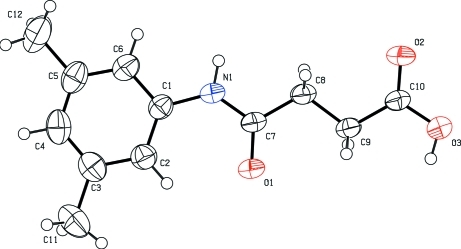
Molecular structure of the title compound, showing the atom labelling scheme. The displacement ellipsoids are drawn at the 50% probability level. The H atoms are represented as small spheres of arbitrary radii.

**Fig. 2.**
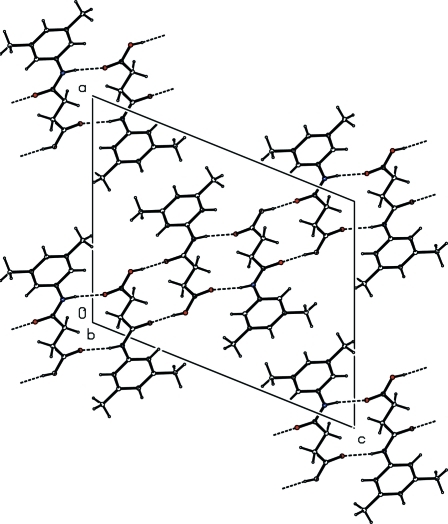
Molecular packing of the title compound with hydrogen bonding shown as dashed lines.

## supplementary crystallographic information

### Comment

As a part of studying the effect of ring and side chain substitutions on the crystal structures of anilides (Gowda *et al.*, 2009, 2010*a*,*b*), in the present work, the crystal structure of *N*-(3,5-dimethylphenyl)- succinamic acid (I) has been determined. The conformations of N—H and C═ O bonds in the amide segment are *anti* to each other (Fig. 1). The conformation of the amide oxygen and the carbonyl oxygen of the acid segment are *anti* to each other, similar to the *anti* conformation observed in *N*-(2,6-dimethylphenyl)-succinamic acid (II) (Gowda *et al.*, 2009), but in contrast to the the *syn* conformation observed in *N*-(3-methylphenyl)succinamic acid (III) (Gowda *et al.*, 2010*a*) and *N*-(3,4-dimethylphenyl)- succinamic acid (IV) (Gowda *et al.*, 2010*b*).

But, the conformations of the amide oxygen and the carbonyl oxygen of the acid segment are *anti* to the adjacent –CH_2_ groups in the above compounds. The conformation of the amide hydrogen is *syn* to one of the *meta*–methyl groups in the benzene ring and *anti* to the other.

Further, the C═O and O—H bonds of the acid group in (I) are in *anti* position to each other, in contrast to the *syn* conformation observed in (II), (III) and (IV).

The intermolecular O—H···O and N—H···O hydrogen bonds pack the molecules into a three-dimensional network (Table 1, Fig. 2).

The modes of interlinking carboxylic acids by hydrogen bonds is described elsewhere (Leiserowitz, 1976). The packing of molecules involving dimeric hydrogen bonded association of each carboxyl group with a centrosymmetrically related neighbor has also been observed (Jagannathan *et al.*, 1994).

### Experimental

The solution of succinic anhydride (0.01 mole) in toluene (25 ml) was treated dropwise with the solution of 3,5-dimethylaniline (0.01 mole) also in toluene (20 ml) with constant stirring. The resulting mixture was stirred for about one h and set aside for an additional hour at room temperature for completion of the reaction. The mixture was then treated with dilute hydrochloric acid to remove the unreacted 3,5-dimethylaniline. The resultant solid *N*-(3,5-dimethylphenyl)-succinamic acid was filtered under suction and washed thoroughly with water to remove the unreacted succinic anhydride and succinic acid. It was recrystallized to constant melting point from ethanol. The purity of the compound was checked by elemental analysis and characterized by its infrared and NMR spectra.

Needle like colorless single crystals used in X-ray diffraction studies were grown in ethanolic solution by slow evaporation at room temperature.

### Refinement

The H atoms of the NH and OH group were located in a difference map and their coordinates were refined. The other H atoms were positioned with idealized geometry using a riding model with C—H = 0.93–0.97 Å. All H atoms were refined with isotropic displacement parameters set to 1.2 times of the *U*_eq_ of the parent atom.

### Crystal data

**Table:**

C_12_H_15_NO_3_	*F*(000) = 472
*M**_r_* = 221.25	*D*_x_ = 1.232 Mg m^−^^3^
Monoclinic, *P*2_1_/*n*	Mo *K*α radiation, λ = 0.71073 Å
Hall symbol: -P 2yn	Cell parameters from 1372 reflections
*a* = 14.346 (2) Å	θ = 2.9–27.7°
*b* = 5.0225 (9) Å	µ = 0.09 mm^−^^1^
*c* = 17.860 (3) Å	*T* = 293 K
β = 112.00 (2)°	Needle, colourless
*V* = 1193.2 (3) Å^3^	0.45 × 0.08 × 0.05 mm
*Z* = 4	

### Data collection

**Table:**

Oxford Diffraction Xcalibur diffractometer with a Sapphire CCD detector	2419 independent reflections
Radiation source: fine-focus sealed tube	1593 reflections with *I* > 2σ(*I*)
graphite	*R*_int_ = 0.019
Rotation method data acquisition using ω scans	θ_max_ = 26.4°, θ_min_ = 3.1°
Absorption correction: multi-scan (*CrysAlis RED*; Oxford Diffraction, 2009)	*h* = −17→17
*T*_min_ = 0.961, *T*_max_ = 0.996	*k* = −6→4
4452 measured reflections	*l* = −22→12

### Refinement

**Table:**

Refinement on *F*^2^	Primary atom site location: structure-invariant direct methods
Least-squares matrix: full	Secondary atom site location: difference Fourier map
*R*[*F*^2^ > 2σ(*F*^2^)] = 0.054	Hydrogen site location: inferred from neighbouring sites
*wR*(*F*^2^) = 0.147	H atoms treated by a mixture of independent and constrained refinement
*S* = 1.03	*w* = 1/[σ^2^(*F*_o_^2^) + (0.0672*P*)^2^ + 0.3965*P*] where *P* = (*F*_o_^2^ + 2*F*_c_^2^)/3
2419 reflections	(Δ/σ)_max_ = 0.018
153 parameters	Δρ_max_ = 0.20 e Å^−^^3^
0 restraints	Δρ_min_ = −0.18 e Å^−^^3^

### Special details

**Table:**

Experimental. CrysAlis RED (Oxford Diffraction, 2009) Empirical absorption correction using spherical harmonics, implemented in SCALE3 ABSPACK scaling algorithm.
Geometry. All e.s.d.'s (except the e.s.d. in the dihedral angle between two l.s. planes) are estimated using the full covariance matrix. The cell e.s.d.'s are taken into account individually in the estimation of e.s.d.'s in distances, angles and torsion angles; correlations between e.s.d.'s in cell parameters are only used when they are defined by crystal symmetry. An approximate (isotropic) treatment of cell e.s.d.'s is used for estimating e.s.d.'s involving l.s. planes.
Refinement. Refinement of *F*^2^ against ALL reflections. The weighted *R*-factor *wR* and goodness of fit *S* are based on *F*^2^, conventional *R*-factors *R* are based on *F*, with *F* set to zero for negative *F*^2^. The threshold expression of *F*^2^ > σ(*F*^2^) is used only for calculating *R*-factors(gt) *etc*. and is not relevant to the choice of reflections for refinement. *R*-factors based on *F*^2^ are statistically about twice as large as those based on *F*, and *R*- factors based on ALL data will be even larger.

### Fractional atomic coordinates and isotropic or equivalent isotropic displacement parameters (Å^2^)

**Table:**

	*x*	*y*	*z*	*U*_iso_*/*U*_eq_	
C1	0.59923 (15)	−0.1845 (4)	0.36154 (12)	0.0429 (5)	
C2	0.56185 (17)	−0.3031 (5)	0.28576 (13)	0.0490 (6)	
H2	0.5005	−0.2483	0.2476	0.059*	
C3	0.61608 (19)	−0.5031 (5)	0.26700 (15)	0.0555 (6)	
C4	0.70735 (19)	−0.5819 (5)	0.32444 (17)	0.0627 (7)	
H4	0.7437	−0.7157	0.3117	0.075*	
C5	0.74574 (18)	−0.4675 (5)	0.40004 (16)	0.0583 (7)	
C6	0.69101 (16)	−0.2678 (5)	0.41832 (14)	0.0506 (6)	
H6	0.7159	−0.1887	0.4691	0.061*	
C7	0.45744 (14)	0.1217 (4)	0.34799 (11)	0.0390 (5)	
C8	0.42783 (15)	0.3396 (4)	0.39303 (12)	0.0419 (5)	
H8A	0.4314	0.2706	0.4448	0.050*	
H8B	0.4759	0.4841	0.4035	0.050*	
C9	0.32429 (15)	0.4476 (4)	0.34847 (12)	0.0477 (6)	
H9A	0.3190	0.5039	0.2951	0.057*	
H9B	0.2757	0.3063	0.3418	0.057*	
C10	0.29831 (15)	0.6774 (4)	0.39033 (11)	0.0417 (5)	
C11	0.5745 (2)	−0.6330 (6)	0.18462 (17)	0.0773 (9)	
H11A	0.5206	−0.5267	0.1489	0.093*	
H11B	0.5499	−0.8074	0.1893	0.093*	
H11C	0.6268	−0.6471	0.1635	0.093*	
C12	0.8446 (2)	−0.5577 (7)	0.46246 (19)	0.0861 (10)	
H12A	0.8325	−0.6906	0.4964	0.103*	
H12B	0.8784	−0.4084	0.4948	0.103*	
H12C	0.8858	−0.6316	0.4359	0.103*	
N1	0.54859 (13)	0.0184 (4)	0.38637 (10)	0.0446 (5)	
H1N	0.5825 (17)	0.090 (5)	0.4335 (14)	0.054*	
O1	0.40041 (11)	0.0430 (3)	0.28096 (9)	0.0557 (5)	
O2	0.35474 (12)	0.7710 (3)	0.45287 (9)	0.0593 (5)	
O3	0.20787 (12)	0.7838 (3)	0.35458 (9)	0.0563 (5)	
H3O	0.1771 (19)	0.695 (5)	0.3110 (16)	0.068*	

### Atomic displacement parameters (Å^2^)

**Table:**

	*U*^11^	*U*^22^	*U*^33^	*U*^12^	*U*^13^	*U*^23^
C1	0.0391 (11)	0.0465 (13)	0.0432 (11)	0.0005 (10)	0.0154 (9)	0.0049 (10)
C2	0.0464 (12)	0.0548 (14)	0.0461 (12)	−0.0005 (11)	0.0178 (10)	−0.0002 (11)
C3	0.0628 (15)	0.0543 (14)	0.0601 (14)	−0.0069 (12)	0.0352 (13)	−0.0027 (12)
C4	0.0632 (16)	0.0578 (15)	0.0810 (19)	0.0093 (13)	0.0429 (15)	0.0059 (14)
C5	0.0473 (13)	0.0631 (16)	0.0691 (16)	0.0074 (12)	0.0271 (12)	0.0175 (13)
C6	0.0446 (12)	0.0580 (14)	0.0477 (12)	0.0015 (11)	0.0158 (10)	0.0075 (11)
C7	0.0361 (10)	0.0416 (12)	0.0323 (10)	−0.0043 (9)	0.0050 (8)	0.0001 (9)
C8	0.0390 (11)	0.0449 (12)	0.0345 (10)	−0.0036 (9)	0.0053 (8)	−0.0026 (9)
C9	0.0436 (12)	0.0479 (13)	0.0375 (11)	0.0041 (10)	−0.0010 (9)	−0.0059 (10)
C10	0.0398 (11)	0.0479 (12)	0.0307 (10)	0.0008 (10)	0.0055 (8)	0.0020 (9)
C11	0.090 (2)	0.0774 (19)	0.0780 (19)	−0.0079 (16)	0.0473 (17)	−0.0227 (16)
C12	0.0632 (17)	0.105 (2)	0.090 (2)	0.0334 (17)	0.0282 (16)	0.0258 (19)
N1	0.0390 (10)	0.0513 (11)	0.0343 (9)	0.0019 (8)	0.0032 (8)	−0.0051 (8)
O1	0.0458 (9)	0.0639 (11)	0.0397 (8)	0.0063 (8)	−0.0042 (7)	−0.0135 (7)
O2	0.0572 (10)	0.0654 (11)	0.0380 (8)	0.0020 (8)	−0.0021 (7)	−0.0156 (8)
O3	0.0492 (9)	0.0673 (11)	0.0414 (8)	0.0137 (8)	0.0043 (7)	−0.0070 (8)

### Geometric parameters (Å, °)

**Table:**

C1—C2	1.389 (3)	C8—H8A	0.9700
C1—C6	1.392 (3)	C8—H8B	0.9700
C1—N1	1.416 (3)	C9—C10	1.496 (3)
C2—C3	1.386 (3)	C9—H9A	0.9700
C2—H2	0.9300	C9—H9B	0.9700
C3—C4	1.385 (3)	C10—O2	1.203 (2)
C3—C11	1.513 (3)	C10—O3	1.325 (2)
C4—C5	1.378 (4)	C11—H11A	0.9600
C4—H4	0.9300	C11—H11B	0.9600
C5—C6	1.386 (3)	C11—H11C	0.9600
C5—C12	1.508 (4)	C12—H12A	0.9600
C6—H6	0.9300	C12—H12B	0.9600
C7—O1	1.235 (2)	C12—H12C	0.9600
C7—N1	1.333 (3)	N1—H1N	0.88 (2)
C7—C8	1.510 (3)	O3—H3O	0.86 (3)
C8—C9	1.499 (3)		
			
C2—C1—C6	119.7 (2)	H8A—C8—H8B	107.7
C2—C1—N1	123.88 (19)	C10—C9—C8	113.35 (17)
C6—C1—N1	116.40 (19)	C10—C9—H9A	108.9
C3—C2—C1	120.0 (2)	C8—C9—H9A	108.9
C3—C2—H2	120.0	C10—C9—H9B	108.9
C1—C2—H2	120.0	C8—C9—H9B	108.9
C4—C3—C2	119.2 (2)	H9A—C9—H9B	107.7
C4—C3—C11	121.1 (2)	O2—C10—O3	119.1 (2)
C2—C3—C11	119.7 (2)	O2—C10—C9	123.95 (19)
C5—C4—C3	121.8 (2)	O3—C10—C9	116.91 (17)
C5—C4—H4	119.1	C3—C11—H11A	109.5
C3—C4—H4	119.1	C3—C11—H11B	109.5
C4—C5—C6	118.6 (2)	H11A—C11—H11B	109.5
C4—C5—C12	121.3 (2)	C3—C11—H11C	109.5
C6—C5—C12	120.1 (3)	H11A—C11—H11C	109.5
C5—C6—C1	120.7 (2)	H11B—C11—H11C	109.5
C5—C6—H6	119.6	C5—C12—H12A	109.5
C1—C6—H6	119.6	C5—C12—H12B	109.5
O1—C7—N1	122.7 (2)	H12A—C12—H12B	109.5
O1—C7—C8	122.14 (18)	C5—C12—H12C	109.5
N1—C7—C8	115.19 (17)	H12A—C12—H12C	109.5
C9—C8—C7	113.56 (16)	H12B—C12—H12C	109.5
C9—C8—H8A	108.9	C7—N1—C1	129.70 (18)
C7—C8—H8A	108.9	C7—N1—H1N	114.7 (15)
C9—C8—H8B	108.9	C1—N1—H1N	115.6 (15)
C7—C8—H8B	108.9	C10—O3—H3O	107.7 (17)
			
C6—C1—C2—C3	−0.1 (3)	N1—C1—C6—C5	179.3 (2)
N1—C1—C2—C3	−179.2 (2)	O1—C7—C8—C9	−0.1 (3)
C1—C2—C3—C4	−0.1 (3)	N1—C7—C8—C9	−179.09 (19)
C1—C2—C3—C11	179.5 (2)	C7—C8—C9—C10	−175.54 (18)
C2—C3—C4—C5	0.2 (4)	C8—C9—C10—O2	1.1 (3)
C11—C3—C4—C5	−179.3 (2)	C8—C9—C10—O3	180.00 (19)
C3—C4—C5—C6	−0.2 (4)	O1—C7—N1—C1	1.5 (4)
C3—C4—C5—C12	179.2 (2)	C8—C7—N1—C1	−179.56 (19)
C4—C5—C6—C1	0.0 (4)	C2—C1—N1—C7	4.9 (4)
C12—C5—C6—C1	−179.4 (2)	C6—C1—N1—C7	−174.2 (2)
C2—C1—C6—C5	0.2 (3)		

### Hydrogen-bond geometry (Å, °)

**Table:**

*D*—H···*A*	*D*—H	H···*A*	*D*···*A*	*D*—H···*A*
N1—H1N···O2^i^	0.88 (2)	2.01 (2)	2.881 (2)	171 (2)
O3—H3O···O1^ii^	0.86 (3)	1.77 (3)	2.630 (2)	172 (3)

Symmetry codes: (i) −*x*+1, −*y*+1, −*z*+1; (ii) −*x*+1/2, *y*+1/2, −*z*+1/2.
